# Antibody and T-Cell Subsets Analysis Unveils an Immune Profile Heterogeneity Mediating Long-term Responses in Individuals Vaccinated Against SARS-CoV-2

**DOI:** 10.1093/infdis/jiac421

**Published:** 2022-10-19

**Authors:** Maria Agallou, Olga S Koutsoni, Maria Michail, Paraskevi Zisimopoulou, Ourania E Tsitsilonis, Evdokia Karagouni

**Affiliations:** Immunology of Infection Laboratory, Hellenic Pasteur Institute, Athens, Greece; Laboratory of Cellular Immunology, Hellenic Pasteur Institute, Athens, Greece; Laboratory of Molecular Neurobiology and Immunology, Hellenic Pasteur Institute, Athens, Greece; Department of Biology, National and Kapodistrian University of Athens, Athens, Greece; Laboratory of Molecular Neurobiology and Immunology, Hellenic Pasteur Institute, Athens, Greece; Department of Biology, National and Kapodistrian University of Athens, Athens, Greece; Immunology of Infection Laboratory, Hellenic Pasteur Institute, Athens, Greece

**Keywords:** COVID-19, antibodies, central memory T cells, cytokines, high and low responders, immune response, stem cell memory T cells, vaccines

## Abstract

**Background:**

Based on the fact that coronavirus disease 2019 (COVID-19) is still spreading despite worldwide vaccine administration, there is an imperative need to understand the underlying mechanisms of vaccine-induced interindividual immune response variations.

**Methods:**

We compared humoral and cellular immune responses in 127 individuals vaccinated with either BNT162b2, mRNA-1273, or ChAdOx1-nCoV-19 vaccine.

**Results:**

Both mRNA vaccines induced faster and stronger humoral responses as assessed by high spike- and RBD-specific antibody titers and neutralizing efficacy in comparison to ChAdOx1-nCoV-19 vaccine. At 7 months postvaccination, a decreasing trend in humoral responses was observed, irrespective of the vaccine administered. Correlation analysis between anti-S1 IgG and interferon-γ (IFN-γ) production unveiled a heterogeneous immune profile among BNT162b2-vaccinated individuals. Specifically, vaccination in the high-responder group induced sizable populations of polyfunctional memory CD4^+^ helper T cells (T_H_1), follicular helper T cells (T_FH_), and T cells with features of stemness (T_SCM_), along with high neutralizing antibody production that persisted up to 7 months. In contrast, low responders were characterized by significantly lower antibody titers and memory T cells and a considerably lower capacity for interleukin-2 and IFN-γ production.

**Conclusions:**

We identified that long-term humoral responses correlate with the individual's ability to produce antigen-specific persistent memory T-cell populations.

## Teaser

A longitudinal study conducted in 2021 revealed that the long-term humoral responses against SARS-CoV-2 detected among BNT162b2-vaccinated individuals correlated with individual's ability to produce persistent antigen-specific T_FH_ and CD4^+^ T-cell memory populations.

The severe acute respiratory syndrome coronavirus 2 (SARS-CoV-2), the etiological agent of coronavirus disease (COVID)-19, has spread worldwide during the last 2 years. As of March 2022, SARS-CoV-2 has infected more than 400 million people and caused about 6 million deaths globally [1]. To achieve a sustainable containment of the pandemic, several vaccines against SARS-CoV-2 have been developed, with mRNA vaccines, namely BNT162b2 and mRNA-1273, being the first approved and administered since December 2020, followed by ChAdOx1-nCoV-19, an adenoviral vectored vaccine. These vaccines conferred protection against COVID-19, with mRNA vaccines having demonstrated higher efficacy and a good safety profile in clinical trials [2–4].

The concentration of produced antibodies against the spike (S) protein or the receptor-binding domain (RBD) and the titers of neutralizing antibodies that prevent binding of SARS-CoV-2 to the angiotensin-converting enzyme 2 (ACE2) receptor, are key measures for evaluating vaccine effectiveness [5–7]. Despite the marked decrease of anti–SARS-CoV-2 antibody levels over time, recent studies have shown that vaccine competency remains high for up to 6 months after initial vaccination [8, 9].

Although older ages have been associated with lower antibody responses [10, 11], there is a subgroup of fully vaccinated young individuals that fails to mount a strong and durable neutralizing antibody response, with no evidence of underlying factors associated with reduced antibody production [12, 13]. Thus, it seems that, besides age and comorbidities, the effectiveness of vaccination depends on factors such as preexisting immunity to the pathogen(s), sex, but also on several unidentified genetic and immune-related factors that impact on antibody response variation. There is evidence that both humoral and cellular immune responses are needed to achieve a robust and persistent protective immunity against SARS-CoV-2 [14–18]. However, the interplay between the 2 arms of adaptive immunity is complex, and their investigation and correlation is difficult to assess. To date, no direct comparison between long-term persistence of humoral and cellular responses has been reported.

Thus, we compared antibody responses in vaccine recipients after the first and second dose of BNT162b2, mRNA-1273, and ChAdOx1-nCoV-19 by conducting a real-life population-based study in Greece. Additionally, assessment of antibody and interferon-γ (IFN-γ) levels at 7 months postvaccination revealed a heterogeneous immune response profile among individuals vaccinated with BNT162b2. Thus, samples obtained from high and low responders were used to identify specific T-cell subsets that likely relate to long-term immunity, with the ultimate goal to identify signatures that can predict the successful outcome of vaccination among individuals.

## METHODS

### Study Population and Ethics Declaration

The present study is a longitudinal study including administrative and laboratory staff of the Hellenic Pasteur Institute (HPI), as well as their family members. Inclusion criteria comprised vaccination against COVID-19, age 18 years or older, and willingness and ability to provide informed consent. Enrolled participants completed a baseline survey questionnaire on demographic data, clinical profile, previous COVID-19 exposure, and vaccine side effects. During the study, participants were subjected to weekly SARS-CoV-2 oropharyngeal swab tests to detect infection. All participants were assigned unique randomization numbers that remained unchanged throughout the study. The study complies with the Declaration of Helsinki and the design of the protocol was approved by the Review Board of the HPI (Ref. No.: 7345/23.06.2021) and the Research Protocol Approval Committee of the Department of Biology, National and Kapodistrian University of Athens (ref. No. 01/21.01.2021).

### Study Design

Sera samples were collected at 6 time points, that is, prior or within 2 days after the first dose (T_0_); 20, 30, or 90 days after BNT162b2, mRNA-1273, and ChAdOx-nCoV-19 first vaccination, respectively (T_1_); and 20 days after the second dose, irrespectively of the vaccine used (T_2_). Additional samples were collected at 3 (T_3_) and 7 months (T_4_) after the second dose and 2 weeks after the third dose (T_5_) given at 5 months up to 10 months after the second dose. At T_0_, T_2_, and T_4_, whole blood was also collected for peripheral blood mononuclear cells (PBMCs) isolation, whereas at T_4_ and T_5_ whole blood was obtained for cytokine quantitation.

### Detection of Anti–SARS-CoV-2 Antibodies

Sera samples were tested for anti-S1 immunoglobulin G (IgG), anti-S1 IgA, and anti-Nucleocapsid Protein (NCP) IgG antibody responses using commercial enzyme-linked immunosorbent assay (ELISA) kits from EuroImmun. cPass SARS-CoV-2 Nabs Detection Kit (Genscript) and SARS-CoV-2-NeutraLISA (EuroImmun) were used for detection of neutralizing antibodies. Anti–spike-RBD IgG, IgG1, IgG2, IgG3, and IgG4 antibodies were measured using a custom ELISA described in Supplementary Material.

### Interferon-γ Release Assay

T-cell responses against SARS-CoV-2 were assessed at T_4_ and T_5_ using an IFN-γ release assay (IGRA; EuroImmun), according to the manufacturer's instructions as described in Supplementary Material. Values > 200 mIU/mL of IFN-γ were considered reactive.

### Cytokine Measurements and Flow Cytometry

The remaining supernatants from the IGRA assay at T_4_ were analyzed by Milliplex MAP Kit using the Human Cytokine/Chemokine Magnetic Bead Panel (EMD Millipore) for interleukin-2 (IL-2), IL-5, IL-13, and tumor necrosis factor-α (TNF-α) according to the manufacturer's instructions. Analysis was performed using a Luminex 200 and data were analyzed using xPONENT software. PBMCs isolated from selected vaccinees at T_0,_ T_2_, and T_4_ were used for assessment of vaccine-induced T-cell immune responses by flow cytometry (Supplementary Material).

### Statistical Analysis

The effect size calculated on G-power analysis estimated a minimum group of n = 120 with a significance level of .05 and a power of 95% covering the BNT162b2 vaccine. For data and statistical analyses, GraphPad Prism version 6.0 was used. Unless specified otherwise, for reporting averaged results, median values were calculated as data contained many outliers and skewed distributions. Tests for statistically significant differences in continuous variables between groups were mainly performed via Mann-Whitney *U* test, unless otherwise specified and adjusted *P* values are displayed. Pairwise correlations were assessed using Spearman rank-order correlation.

## RESULTS

### Characteristics of the Study Cohort

A total of 127 participants were included in the final analysis (Supplementary Table 1). Among enrolled individuals, 64.57% were female and 35.43% male with a mean age ± SD of 46.10 ± 13.38 years. In general, 67.72% of participants were self-reported as healthy, while 32.28% reported at least 1 comorbidity and 37.01% mentioned outpatient self-medication. Among them, 16.53% had an autoimmune disease, followed by asthma (8.66%) and arterial hypertension (5.51%) (Table 1). Comparing the number of total adverse events after the first and the second dose of each vaccine, reactogenicity after the second dose was significantly higher in individuals receiving BNT162b2 and mRNA-1273, while the opposite pattern was observed for individuals receiving the ChAdOx1-nCoV-19 vaccine (Supplementary Table 2). The vast majority of symptoms were mild to moderate in terms of severity, coinciding with published reports on vaccine safety [19].

**Table 1.** Key Baseline Demographic and Clinical Characteristics of Study Participants

Variable	BNT162b2 (n = 102)	mRNA-1273 (n = 14)	ChAdOx1-S (n = 11)	Total (n = 127)
Sex				
ȃFemale	72/102 (70.59)	4/14 (28.6)	6/11 (54.55)	82/127 (64.57)
ȃMale	30/102 (29.41)	10/14 (71.4)	5/11 (45.45)	45/127 (35.43)
Age group, y				
ȃYoung adults, total	37/102 (36.28)	2/14 (14.3)	4/11 (36.4)	43/127 (33.86)
ȃ 18–30	18/102 (17.65)	2/14 (14.3)	1/11 (9.1)	21/127 (16.54)
ȃ 31–40	19/102 (18.63)	0/14 (0.0)	3/11 (27.3)	22/127 (17.32)
ȃMiddle aged, total	51/102 (50.0)	7/14 (50.0)	1/11 (9.1)	59/127 (46.46)
ȃ 41–50	32/102 (31.37)	3/14 (21.4)	1/11 (9.1)	36/127 (28.35)
ȃ 51–60	19/102 (18.63)	4/14 (28.6)	0/11 (0.0)	23/127 (18.11)
ȃOlder adults (> 60)	14/102 (13.72)	5/14 (35.7)	6/11 (54.5)	25/127 (19.68)
Mean age, y ± SD	45.22 ± 12.6	53.1 ± 16.0	50.5 ± 14.0	46.10 ± 13.38
Mean bodyweight, kg ± SD	73.76 ± 19.9	81.2 ± 11.6	81.4 ± 16.8	74.36 ± 19.14
Mean height, cm ± SD	169.37 ± 8.8	173.6 ± 7.2	173.5 ± 10.1	170.37 ± 8.86
Mean BMI, kg/m^2^ ± SD	25.45 ± 5.6	26.9 ± 3.0	26.9 ± 4.3	25.44 ± 5.26
Comorbidity				
ȃAsthma	10/102 (9.80)	0/14 (0.00)	1/11 (9.09)	11/127 (8.66)
ȃArterial hypertension	6/102 (5.88)	1/14 (7.14)	0/11 (0.00)	7/127 (5.51)
ȃAutoimmune disease	19/102 (18.63)	0/14 (0.00)	2/11 (18.18)	21/127 (16.53)
ȃOther	10/102 (9.80)	0/14 (0.00)	1/11 (9.09)	11/127 (8.66)
COVID-19 infection				
ȃPast infection^a^	1/102 (0.98)	0/14 (0.00)	0/11 (0.00)	1/127 (0.79)
ȃPostinfection^b^	8/102 (7.84)	0/14 (0.00)	0/11 (0.00)	8/127 (6.30)
Blood type				
ȃ0	31/102 (30.39)	6/14 (42.86)	0/11 (0.00)	37/127 (29.13)
ȃA	41/102 (40.20)	6/14 (42.86)	7/11 (63.64)	54/127 (42.52)
ȃB	8/102 (7.84)	1/14 (7.14)	0/11 (0.00)	9/127 (7.09)
ȃAB	2/102 (1.96)	0/14 (0.00)	0/11 (0.00)	2/127 (1.57)
ȃUnknown	20/102 (19.61)	1/14 (7.14)	4/11 (36.36)	25/127 (19.69)
Rhesus				
ȃNegative	12/102 (11.76)	0/14 (0.00)	0/11 (0.00)	12/127 (9.45)
ȃPositive	68/102 (66.67)	12/14 (85.71)	8/11 (72.73)	88/127 (69.29)
ȃUnknown	22/102 (21.57)	2/14 (14.29)	3/11 (27.27)	27/127 (21.26)
Other vaccines in the past year				
ȃInfluenza	44/102 (43.14)	5/14 (35.71)	5/11 (45.45)	54/127 (42.52)
ȃPneumococcal	12/102 (11.76)	3/14 (21.43)	2/11 (18.18)	17/127 (13.39)
ȃHerpes simplex virus	0/102 (0.00)	0/14 (0.00)	0/11 (0.00)	0/127 (0.00)
Outpatient self-medicated				
ȃYes	40/102 (39.22)	2/14 (14.29)	5/11 (45.45)	47/127 (37.01)
ȃNo	62/102 (60.78)	12/14 (85.71)	6/11 (54.55)	80/127 (62.99)

Data are frequency (%) except where indicated.

Individuals that have been infected prior vaccination.

Individuals that have been infected post vaccination.

### Comparative Kinetic Analysis of Humoral Responses Among the Different Vaccines

**Figure 1.**
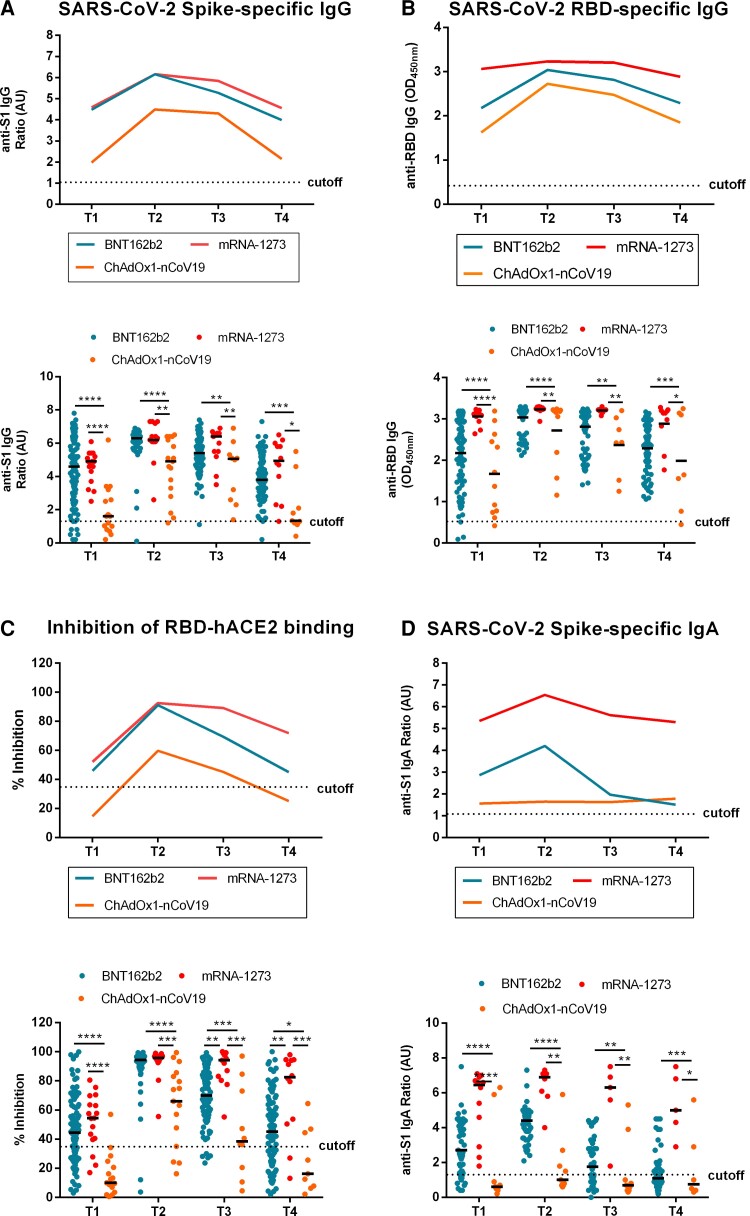
Kinetic analysis of antibody responses and neutralization activity after vaccination with BNT162b2, mRNA-1273, and ChAdOx1-nCoV-19. Serum samples from BNT162b2-, mRNA-1273- and ChAdOx1-nCoV-19-vaccinated individuals were collected at 20, 30, and 90 days after the priming dose (T_1_), respectively. Serum was also collected at 20 days (T_2_), 3 months (T_3_), and 7 months (T_4_) after the second dose, irrespectively of the administered vaccine. Average and individual values of (*A*) anti-spike IgG titers (ratio), (*B*) anti-RBD IgG (OD_450_), (*C*) neutralization activity (% inhibition), and (*D*) anti-spike IgA titers (ratio); SD values are not shown for clarity. In individual values of anti-spike IgG, anti-RBD IgG, anti-spike IgA, and neutralization activity each dot represents 1 participant. Horizontal lines indicate median values. Comparison between groups was performed by multiple 2-paired Student *t* test and statistical significance was assessed using Holm-Sidak method. * *P* < .05, **P < .01, ***P < .001, ****P < .0001.

Primary vaccination with BNT162b2 induced detectable anti-S1 IgG, anti-RBD IgG, and anti-S1 IgA antibody responses in 94.2% of vaccine recipients (ratio >1.1), while 66.7% exhibited an intermediate neutralization activity of 44.55% (>35%) (Figure 1). Boosting with the second dose (T_2_) led to the enhancement of all antibodies tested in 98% of the participants and to an impressive increase of neutralization activity (Figure 1). At T_3_, all antibodies remained at high levels despite a slight decline, and a similar trend was recorded for neutralizing antibodies (Figure 1). Eventually, at T_4_, anti-S1 IgG and anti-S1 IgA decreased by about 2-fold with a median neutralization activity of 45.30% (Figure 1). Among study participants, 2 were previously infected with SARS-CoV-2 and their antibodies produced after the first dose were at similar levels to those detected at T_2_ in the rest of the population, which remained stably elevated up to 7 months (T_4_). On the contrary, severely immunocompromised individuals did not mount a humoral immune response at any time point.

mRNA-1273–vaccinated individuals displayed an impressively uniform and homogeneous pattern compared to BNT162b2 (Figure 1). Specifically, at T_1_, 100% of participants produced high anti-S1 IgG, anti-RBD IgG, and anti-S1 IgA levels, and 75% developed a strong neutralizing activity. Moreover, at T_2_, all antibody levels, as well as median neutralizing activity, significantly increased, plateaued until T_3_, and slightly declined at T_4_ with only 1 participant turning marginally negative (Figure 1).

ChAdOx1-nCoV-19 vaccine recipients exhibited a quite different profile from both mRNA-vaccinated participants. Specifically, 37.5% of participants were negative for anti-S1 IgG antibodies at T_1_, while 62.5% of anti–S1-positive participants did not develop neutralizing antibodies before the second dose (Figure 1), revealing a slow antibody production independent of age or sex (Supplementary Figure 2). The booster dose enhanced anti-S1 IgG and anti-RBD IgG antibody levels in all participants with only 70.6% being capable of neutralization with a median value of 66.0% (Figure 1). The same effect was observed at T_3_ followed by significant reduction at T_4_ with 22.2% of participants being negative for anti-S1 IgG and 66.7% for neutralizing antibodies (Figure 1). Importantly, ChAdOx1-nCoV-19 vaccinees were found to be negative for anti-S1 IgA antibodies (Figure 1).

Overall, ChAdOx1-nCoV-19 was less effective compared to the mRNA vaccines, whereas mRNA-1273 was slightly advantageous over BNT162b2 in terms of antibody production. However, in all cases, results showed strong correlations between all antibody measurements at all time points (T_1_–T_4_) (Supplementary Figure 3). Age-associated differences in neutralization activity were detected for BNT162b2 after the first and second vaccinations (Supplementary Figure 4), whereas, despite the limited sample size, sex-associated differences were identified in ChAdOx1-nCoV-19 vaccine recipients, with women being less responsive compared to men (Supplementary Figure 5).

### Evaluation of S1-Specific IFN-γ Responses Induced by the 3 Vaccines

**Figure 2.**
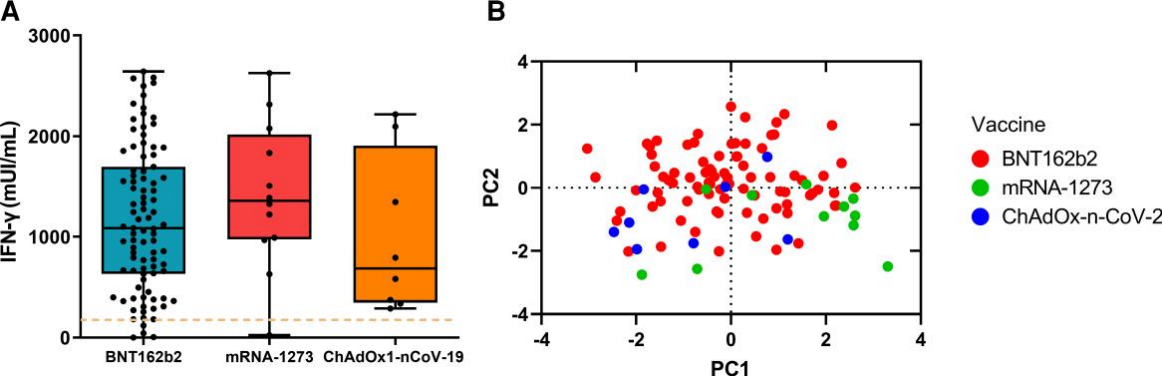
*A*, Interferon-γ (IFN-γ) production, assessed by IFN-γ release assay, in whole blood cells from vaccinated participants at 7 months after the second dose. Each dot represents an individual in all plots. Boxes show median and 25th–75th percentiles; whiskers show the range in all box plots. The statistical difference between the 3 vaccines was calculated using 2-sided Mann-Whitney rank-sum test. *B*, Principal component (PC) analysis of antibody and IFN-γ responses in vaccinated participants at 7 months after the second dose.

Based on the fact that at 7 months after the second vaccination (T_4_), a notable number of vaccine recipients exhibited a significant decline of neutralizing antibodies, we determined the presence of S1-specific cellular immune responses by assessing the production of IFN-γ. We found that 94.5% of BNT162b2 and 91.6% of mRNA-1273 vaccine recipients produced IFN-γ above threshold and at similar levels, with a median production of 1086.0 mUI/mL and 1357.0 mUI/mL, respectively (Figure 2*A*). Regarding ChAdOx1-nCoV-19, 100% of recipients produced 2-fold lower levels of IFN-γ compared to those detected in mRNA vaccine recipients (Figure 2*A*). Using Spearman test, no correlation was found between secreted IFN-γ and neutralizing antibody levels for all tested vaccines. Principal component analysis mapping using the 7-month postvaccination data revealed a rather heterogenic response among BNT162b2 recipients in contrast to mRNA-1273 and ChAdOx1-nCoV-19 vaccine recipients that showed a more homogenous distribution based on anti-S1 IgG production, neutralization activity, and IFN-γ secretion (Figure 2*B* and Table 2).

**Table 2.** Long-term Spike S1-Specific Humoral and Cellular Immune Responses in Individuals Vaccinated With BNT162b2

Variable^a^	High Antibody-High IFN-γ Group	Low Antibody-High IFN-γ Group	High Antibody-Low IFN-γ Group	Low Antibody-Low IFN-γ Group
Neutralization activity, % inhibition^a^	62.2 (53.2–74.1)	23.9 (15.3–33.7)	58.3 (49.2–75.8)	24.2 (15.3–32.9)
IFN-γ, mUI/mL^b^	1632.0 (1343.0–2117.0)	1815.0 (1503.0–2226.0)	635.5 (308.6–720.2)	578.9 (339.5–956.8)

Data are median values (interquartile ranges).

Abbreviation: IFN-γ, interferon-γ.

A value of < 30% inhibition is considered negative.

A value of < 200mUI/mL is considered negative.

### Comparative Analysis of the Underlying Cellular Responses

To identify the factors that are related to the distinct immunological profiles detected in the BNT162b2-vaccinated individuals, the 2 groups found at the extremes of antibody and cytokine responses, that is, high antibody-high IFN-γ (HH) and low antibody-low IFN-γ (LL) (Table 2) were selected for the determination of phenotypic traits and cytokine expression patterns. It must be noted that the size and median age of the 2 groups were similar. Convalescent individuals or participants on immunosuppressive medication were excluded, because their immune responses would be biased by infection or medication. Moreover, anti-NCP IgG antibody detection in the HH group throughout the study period excluded any asymptomatic infection (Supplementary Figure 6).

**Figure 3.**
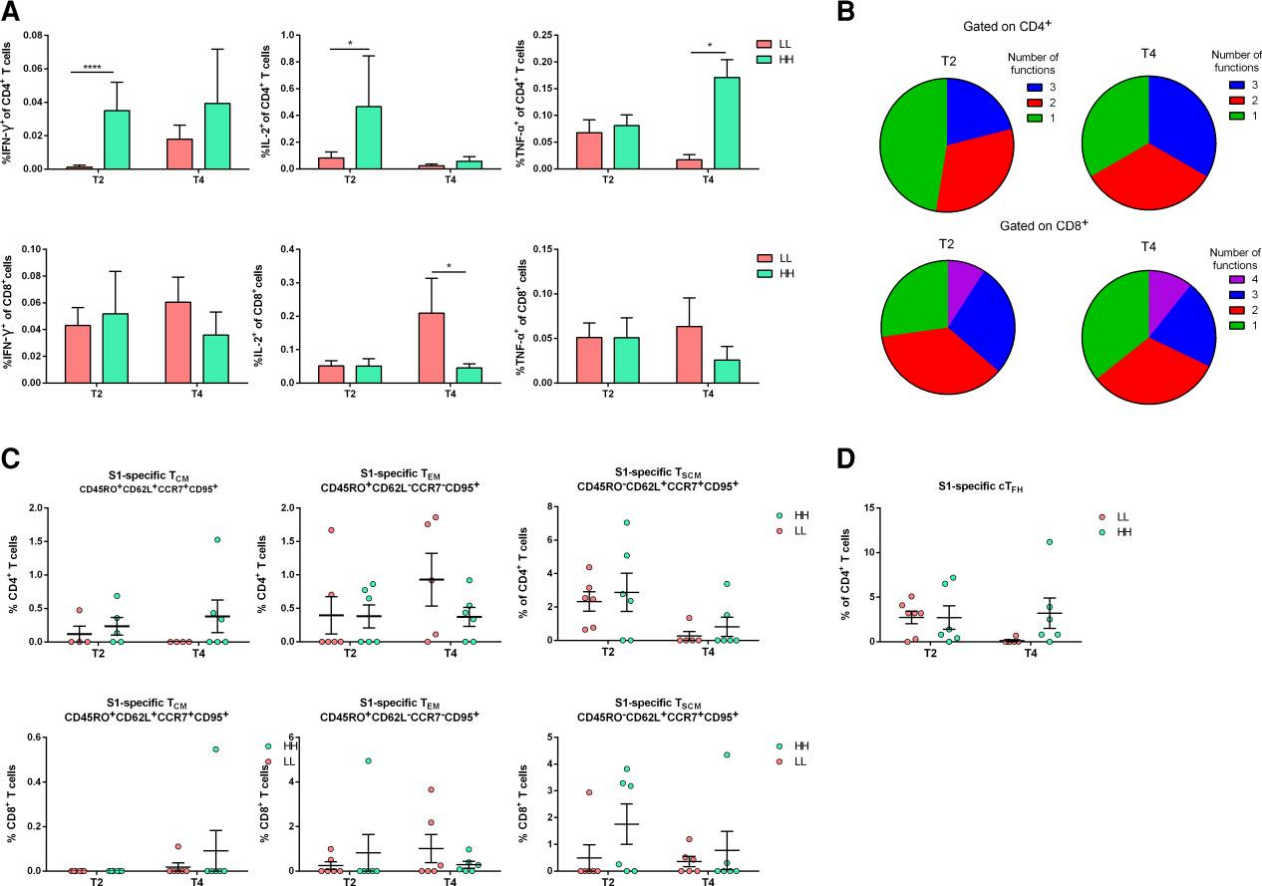
Analysis of S1-specific CD4^+^ and CD8^+^ T-cell subsets in low (LL) and high (HH) responders at 20 days (T_2_) and 7 months (T_4_) after second vaccination. *A*, Frequencies of S1-specific IFN-γ, IL-2, and TNF-α–producing CD4^+^ and CD8^+^ T-cell subsets. Bar graphs show mean values ± SEM. *B*, Polyfunctional analysis and relative distribution of single or multiple cytokine responses in CD4^+^ and CD8^+^ T-cell subsets. *C*, Frequencies of central memory (CM; CD45RO^+^CD62L^+^CCR7^+^CD95^+^), effector memory (EM; CD45RO^+^CD62L^−^CCR7^−^CD95^+^), and stem cell memory (SCM; CD45RO^−^CD62L^+^CCR7^+^CD95^+^) in CD4^+^ and CD8^+^ T cells. *D*, Frequencies of follicular helper (FH; CD4^+^CXCR5^+^) T cells. Each dot represents 1 participant. Horizontal lines indicate mean values ± SEM. The statistical difference between the 2 groups was calculated using 2-sided Mann-Whitney rank-sum test. * *P* < .05, ****P < .0001.

To investigate the cellular immune responses in depth, intracellular cytokine staining (ICS) was performed in PBMCs isolated at T_2_ and T_4_ from 6 individuals from each group after stimulation with spike N-terminal S1 and C-terminal S2. After the boost dose, all individuals responded to S1 and S2 stimulation with ICS^+^CD4^+^ and ICS^+^CD8^+^ T cells (Figure 3*A* and Supplementary Figure 8*A*). Importantly, the HH group acquired higher frequencies of CD4^+^IFN-γ^+^ and CD4^+^IL-2^+^ as compared to the LL group against S1 (Figure 3*A*). Also, CD4^+^IFN-γ^+^ remained largely unaffected followed by a CD4^+^TNF-α^+^ T-cell increase at T_4_ (Figure 3*A*). No significant differences were found regarding ICS^+^CD8^+^ T cells among HH and LL groups, which were maintained to 80% of HH and LL responders at T_4_ (Figure 3*A*). Nonetheless, the responses against S2 were equal among the 2 groups in CD4^+^ T cells populations, whereas LL responders were superior in CD8^+^ T cells producing IFN-γ, IL-2 and TNF-α (Supplementary Figure 8A). Multifunctional analysis revealed that 80% of HH individuals contained significantly enhanced frequencies of IFN-γ^+^TNF-α^+^, IFN-γ^+^IL-2^+^ and TNF-α^+^IL-2^+^ T cells against S1, with the latter 2 subgroups remaining detectable at T_4_. Regarding CD8^+^ T cells, the LL group contained higher frequencies of IFN-γ^+^TNF-α^+^ T cells against S1 as well as CD107a^+^IL-2^+^ and CD107a^+^TNF-α^+^ against S1 and S2, which remained stable until T_4_ (Figure 3*B*, Supplementary Figure 8*A* and Supplementary Figure 9).

Detection of major T-cell subsets revealed that prevaccination responses were undetectable in the majority of individuals, although some of them had low frequencies of antigen-specific T cells (mainly against S2 domain) irrespective of their vaccination group, which may be attributed to cross-reactive cells from prior seasonal coronavirus infection (Supplementary Figure 10). Vaccination induced S1- and S2-specific T follicular helper (T_FH_) cells in 83.3% of HH and LL individuals and 100% and 83.3% of HH and LL, respectively. Importantly, S1-specific T_FH_ were still detected in 83% of vaccinees of the HH group in contrast to 16.7% of the LL group at T_4_, whereas S2-specific T_FH_ were maintained up to 7 months in LL group (Figure 3*D* and Supplementary Figure 8*C*). Regarding memory T-cell subsets, it was found that effector memory cells against S1 and S2 dominated CD4^+^ and CD8^+^ T-cell subsets in both HH and LL groups, with similar frequencies at T_2_ and a tendency to increasing numbers at T_4_. Regarding central memory populations (T_CM_), only the HH group exhibited high frequencies in the CD4^+^ T-cell subset when stimulated with S1, which remained stable until T_4_ (Figure 3*C*). Importantly, a significant number of CD4^+^ and CD8^+^ T cells with stem cell memory (T_SCM_) phenotype was detected in the HH group at T_2_, specific for S1 and S2 and higher than that observed in the LL group. T_SCM_ cell subsets specific for S1 were preserved at significant numbers at T_4_ (Figure 3*C* and Supplementary Figure 8*C*).

**Figure 4.**
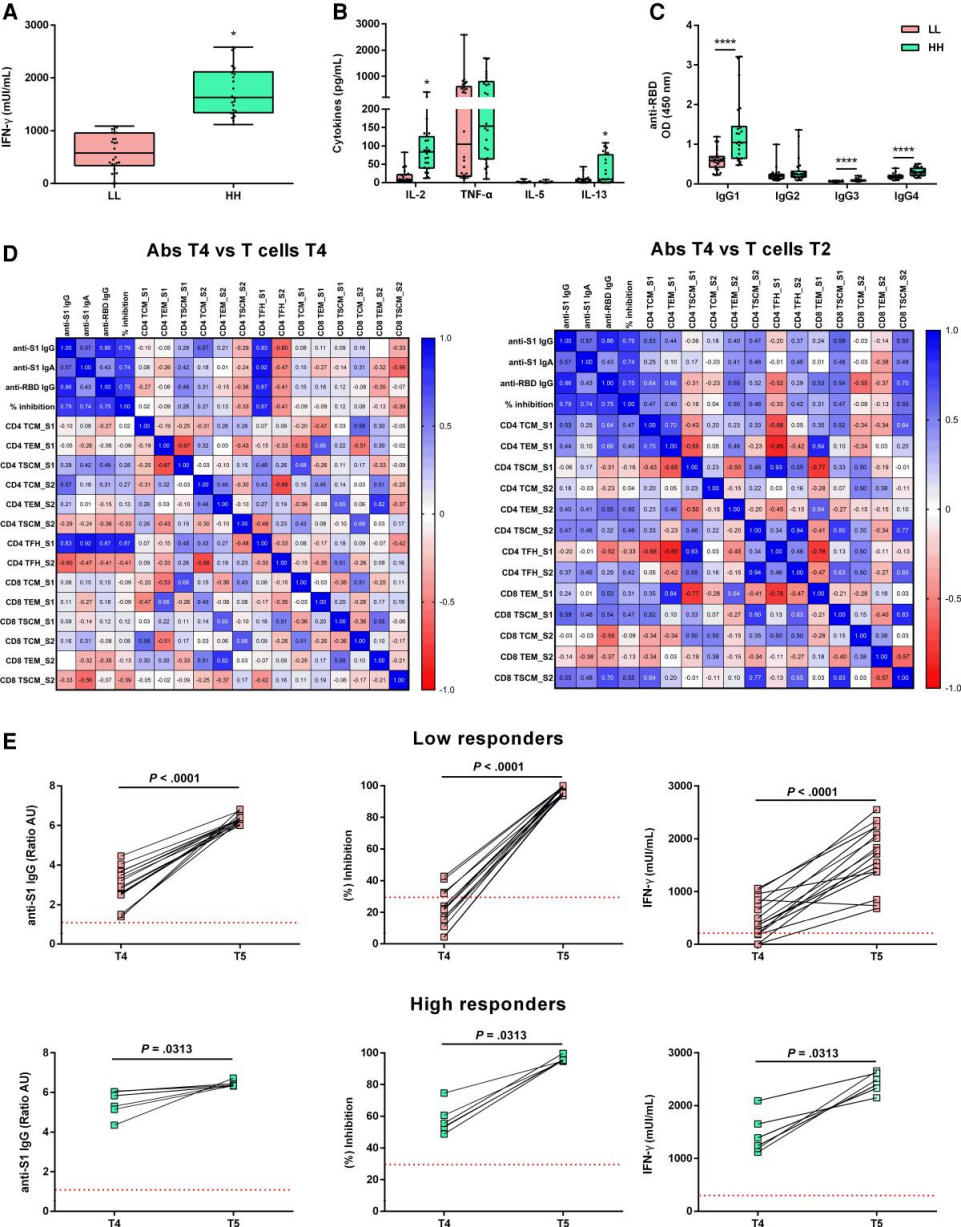
Assessment of (*A***)** IFN-γ and (*B*) IL-2, TNF-α, IL-5, and IL-13 production after S1-peptide restimulation of whole blood cells, and (*C*) anti-RBD IgG1, IgG2, IgG3, and IgG4 subclasses in sera obtained from BNT162b2-vaccinated participants in the LL and HH groups. Each dot represents an individual in all plots. Boxes show median and 25th–75th percentiles; whiskers show the range in all box plots. Statistical differences between groups were calculated using 2-sided Mann-Whitney rank-sum test. *P < .05, ****P < .0001. *D*, Correlation matrix heatmap of S1- and S2-specific humoral and cellular immune responses. Spearman correlation coefficient is shown. *E*, Immune responses of HH and LL responders after the third dose of the BNT126b2 vaccine. Comparison of anti-S1 IgG antibodies levels (ratio), neutralizing activity (% inhibition), and IFN-γ levels after S1-peptide restimulation of whole blood cells at 7 months after second vaccination (T_4_) and 2 weeks after third vaccination (T_5_) are shown. Lines connect samples from the same individual. Statistical differences between the 2 time points were calculated using 2-sided Mann-Whitney rank-sum test. Abbreviations: Abs, antibodies; HH, high antibody-high IFN-γ; IFN-γ, interferon-γ; IgG, immunoglobulin G; IL, interleukin; LL, low antibody-low IFN-γ; OD, optical density; RBD, receptor-binding domain; TCM, central memory T cell; TEM, effector memory T cell; TFH, follicular helper T cell; TNF-α, tumor necrosis factor-α; TSCM, stem cell memory T cell.

High responders are characterized by an overall T_H_1-type cytokine secretion profile. T_H_1 versus T_H_2-type cytokine secretion profile was determined by detection of IL-2, TNF-α, IL-5, and IL-13 levels after stimulation of whole blood with spike S1 domain. The HH group responded to S1 peptide restimulation by producing 3-fold higher IFN-γ and 10-fold higher IL-2 compared to the LL group (Figure 4*A*, Figure 4*B* and Table 3). Surprisingly, no differences were detected in TNF-α levels between high and low responders, as well as in IL-5, which in most cases was marginally detectable. On the contrary, IL-13 levels were significantly higher (4.5-fold) in the HH group compared to the LL group (Figure 4*B* and Table 3). Spearman correlation analyses between individual S1-specific cellular and humoral immune responses revealed a positive significant correlation between IFN-γ and IL-2, IFN-γ and IL-13, IL-13 and IL-2, as well as IFN-γ and TNF-α (Supplementary Figure 11). Also, significant correlation among all cytokines tested and neutralization activity was observed (Supplemenary Figure 12). In parallel, anti-RBD IgG subclass analysis revealed a superior production of IgG1 antibodies in the HH group relative to T_H_1 profile (Figure 4*C*). Spearman correlation analyses considering all parameters of vaccine-induced immune responses at T_4_ revealed a significant association between antibody levels and S1-specifc T_FH_ cells and CD4^+^ T_SCM_ cells (Figure 4*D*). Moreover, correlation analysis between T-cell subsets at T_2_ and antibody responses at T_4_ revealed a strong association of antibodies with S1-specific memory CD4^+^ T cells, providing an indicator of long-term humoral immunity (Figure 4*D*).

**Table 3.** Levels of Secreted Cytokines in the High Antibody-High IFN-γ and Low Antibody-Low IFN-γ Groups of Individuals Vaccinated With BNT162b2

Variable	High Antibody-High IFN-γ Group	Low Antibody-Low IFN-γ Group
IFN-γ, mUI/mL	1632.0 (1343.0–2117.0)	578.9 (339.5–956.8)
IL-2, pg/mL	83.3 (39.8–125.4)	8.1 (3.5–21.3)
TNF-α, pg/mL	154.1 (64.2–802.5)	104.6 (17.6–606.3)
IL-5, pg/mL	0.6 (0.3–1.2)	0.3 (0–1.6)
IL-13, pg/mL	8.9 (0–75.7)	1.9 (0–8.4)

Data are median values (interquartile ranges). Cytokines are defined as the participant-specific S1-stimulated responses minus the unstimulated response.

Abbreviations: IFN-γ, interferon-γ; IL, interleukin; TNF-α, tumor necrosis factor-α.

Assessment of antibody responses along with IFN-γ production in the HH and LL groups, 2 weeks after the third dose (T_5_) showed that the LL group exhibited a 2.1-fold increase in median anti-S1 IgG titer and a 4.4-fold increase in median neutralization activity (median value 97.1%), reaching the levels detected in the HH group (Figure 4*E*). Regarding cellular immune responses, the LL group responded to S1 peptide restimulation by producing 4.6-fold increased levels of IFN-γ as compared with those detected at T_4_, reaching the levels detected in HH group (Figure 4*E*).

## DISCUSSION

The development of multiple vaccines is one of the key pillars of humanity's eventual success against the COVID-19 pandemic. Authorized vaccines, despite their differences in technology used, provide significant protection against SARS-CoV-2 infection [2–4]. Vaccination-induced neutralizing antibodies are considered vital correlates of protection, because they have been consistently associated with prevention of symptomatic disease [5, 7]. In accordance with previous reports [20, 21], our findings showed that BNT162b2 and mRNA-1273 effectively mobilized robust humoral immune responses in healthy, as well as in convalescent, individuals after the first dose, in contrast to ChAdOx1-nCoV-19 that required 2 doses. In general, irrespective of the vaccine administered, antibody responses were maintained for up to 7 months, with only 1 documented symptomatic infection, suggesting that neutralizing titers can also be used as surrogate markers of vaccine efficacy. Previous studies showed both anti-S1 IgG and neutralizing antibody persistence for at least 6 months following BNT162b2 and mRNA-1273 vaccination [21–23]. In our cohort, antibody levels exhibited a gradual decrease at 3 months after the second dose, with 30% of participants having lost their neutralization activity at 7 months in all vaccines tested (T_4_ in Figure 1). Likewise, several groups have reported a drop in antibody titers along with a marked decrease in neutralizing capacity in the long term [22, 24–26]. Nevertheless, in our study, the reduction observed at T_4_ was age independent, in contrast to the documented inverse relationship between age and neutralizing responses after the first dose of BNT162b2 and mRNA-1273 [10, 27]. Specifically, we found a striking interindividual variation in the amplitude and nature of the humoral response explained only in part by age, sex, previous exposure, and drug treatments.

In many cases, waning of antibodies in peripheral blood does not necessarily associate with the absence of specific protection against SARS-CoV-2, because it has been demonstrated that virus-specific memory B cells persist for more than 240 days after COVID-19 symptom onset [28, 29]. Memory B-cell activation and eventual antibody production are supported by the presence of antigen-specific cell responses, which are not necessarily dependent on follicular T cells [30, 31]. Thus, the generation of adequate antigen-specific T-cell responses aids memory B-cell activation and, eventually, antibody production. This is similar to the responses induced by the hepatitis B vaccine, where no cases of acute hepatitis B or chronic antigen carriage have been reported, despite the failure of the vaccine to generate strong antibody response even after the booster dose [32, 33]. Evaluation of SARS-CoV-2–specific cell-mediated immune responses unveiled a high heterogeneity in their magnitude among BNT162b2-vaccinated participants, which was not related to anti-S1 IgG and neutralizing efficacy, in contrast to mRNA-1273 and ChAdOx1-nCoV-19 vaccine recipients, as also previously reported [22]. Unimpaired vaccine-specific humoral and cellular responses have been reported in tick-borne encephalitis, hepatitis B, and smallpox vaccination, in which cases efficacy depended not only on the vaccine antigen but also on the genetic predisposition of vaccinated individuals [34, 35].

This heterogeneity prompted us to group BNT162b2-vaccinated participants with similar immune responses in 2 immune extreme phenotypes, that is, high or low levels of both humoral and cellular responses, further designated as high and low responders, respectively. The main characteristics of high responders were the significantly increased numbers of S1-specific CD4^+^ T_CM_ and T_SCM_ with a multifunctional profile, as well as T_FH_ cells that were maintained up to 7 months postvaccination, which in many cases guarantee vaccination success, because both are associated with superior pathogen control via establishment of T_SCM_-mediated long-lived immunity [36–38]. This is verified in our study by the significant correlation between all memory populations and the neutralizing activity of anti-S1–specific antibodies. Indeed, several groups have shown that extensive IgG class switching is probably instructed by vaccine-induced T_H_1-polarized CD4^+^ T-cell responses [39–41]. Moreover, the importance of high IL-2 levels, which indirectly stimulate B cells via T_H_-cell differentiation, was also evidenced in vaccinated or COVID-19 as well as in SARS-CoV-1 convalescent individuals [42–45].

Our most interesting finding was that about 30% of high responders were capable of producing significantly high levels of IL-13, a signature-cytokine produced by T_H_2 cells [46]. In vitro data have shown that IL-13 induces the proliferation and differentiation of human B cells [47], and this is in agreement with the high neutralizing activity of high responders detected at 7 months postvaccination. Despite the fact that data regarding COVID-19 so far have linked IL-13 production with disease severity [48], a recent study on vaccine-induced immune responses in immunocompromised and healthy individuals revealed that booster vaccinations induced memory T-cell populations able to produce not only T_H_1 skewed cytokines, but also high levels of IL-13 [49]. The supportive role of IL-13 in B-cell activation and eventually in antibody production was further supported in our study by the significant increase of humoral immune responses along with IFN-γ production, detected in IL-13 high responders after the third dose of the vaccine. Similarly, high responders to hepatitis B vaccination were capable of producing high levels of IL-13 after antigenic stimulation, which were significantly correlated with plasma IgG levels, suggesting that the levels of IL-13 are involved in the determination of antigen-specific memory B-cell number [50].

In conclusion, by using immune extreme phenotypes we were able to provide a deeper insight into vaccine responses by explaining and characterizing interindividual differences in both antibody and cellular responses. Specifically, we demonstrated that the induction of high numbers of antigen-specific T_FH_ and CD4^+^ T-cell memory populations, able to produce high levels of IL-2, IFN-γ, and in some cases of IL-13, are positively correlated with increased and sustained long-term antibody responses.

## Supplementary Data

Supplementary materials are available at *The Journal of Infectious Diseases* online. Consisting of data provided by the authors to benefit the reader, the posted materials are not copyedited and the sole responsibility of the authors, so questions or comments should be addressed to the corresponding author.

## Notes

***Acknowledgments***. We thank Athena Tzifa, Athanasia Zavou, Evaggelia Paliou, and Dr Antonia Efstathiou for technical assistance in whole blood and sera sampling and Dr Maritsa Margaroni for technical assistance in flow cytometry.

***Disclaimer***. The funders had no role in study design, data collection, data analysis, interpretation, writing, or submission of the manuscript.

***Financial support***. This work was supported by the Bank of Greece, the Bodossaki Foundation and the Regional Operational Programme of Attica (MIS 5066768).

## Supplementary Material

jiac421_Supplementary_Data
