# Supplementary material for: Antibody and T-Cell Subsets Analysis Unveils an Immune Profile Heterogeneity Mediating Long-term Responses in Individuals Vaccinated Against SARS-CoV-2
Source: J Infect Dis. 2022 Oct 19;227(3):353–63. doi: 10.1093/infdis/jiac421 (PMC9620767; doi:10.1093/infdis/jiac421)
Supplement: jiac421_Supplementary_Data [file jiac421_supplementary_data.zip › Agallou_Maria_Supplementary data_Version_2.docx]

**Supplementary Data**

**Data collection and questionnaires**

Enrolled participants were firstly informed about the purpose of the study and after providing their consent to participate, they completed a baseline survey questionnaire that was constructed based on a modified version of the Vaccine Adverse Event Reporting System ([www.vaers.hhs.gov](http://www.vaers.hhs.gov)) and on other related studies [1, 2, 3]. The questionnaire included four main parts, in the following sequence: (a) demographic data including age, sex, height, weight and blood type; (b) clinical profile including comorbidities, such as respiratory abnormalities, heart and autoimmune diseases, and medication; (c) COVID-19 related anamnesis including vaccination date, vaccine brand, number of doses, previous infection, and exposure to infected cases; and (d) vaccine side effects including local and systemic adverse reactions at the indicated time-points surveys (after each vaccination). Response options were selected according to the reported adverse events in vaccine clinical trials. Moreover, participants reported receipt of influenza, pneumococcal or herpes simplex virus (HSV) shots in the past year.

**Statistical analysis**

The obtained results were analyzed with the Statistical Package for the Social Sciences (SPSS) software version 23 (Chicago, IL, USA). Descriptive statistics including means, standard deviation, median, absolute frequencies and percentages were used to describe baseline demographic and clinical characteristics, and responses for adverse events. Student’s t-test, Fisher's exact and Chi-square tests were used to find statistically significant differences and the associated risk factors for developing symptoms after vaccination. The level of significance was declared based on odd ratio (OR) of 1 with a confidence level of 95% and a P value of ≤ 0.05.

**Key baseline demographic and clinical characteristics of study participants**

A total of 127 participants filled in the questionnaire and were included in the final analysis. The enrolled participants reported being vaccinated according to the national COVID-19 vaccination schedule and they received a total of 297 doses. In more detail, 102 (80.31%) of individuals had completed the primary vaccination series of the BNT162b2 vaccine, by receiving two vaccine shots and 36 of them also received the third-dose boost. 14 (11.03%) participants had received two doses of the mRNA-1273 vaccine, while 11 (8.66%) received two doses of the ChAdOx1-S vaccine, of whom 7 received also the third-dose boost (Supplementary Table 1).

Participants were divided into three age groups that covered young adulthood (18 to 40 years), middle age (41 to 60 years) and older adulthood (older than 60 years). 43 (33.86%) of participants were in the young age group, while 59 (46.46%) and 25 (19.68%) were in the middle age and old age group, respectively. The age of all participants ranged between 21 and 75 years, with a median of 45 years. Mean age was 45.22 ± 12.6 years in the BNT162b2 vaccine group, 53.1 ± 16 years in the group vaccinated with mRNA-1273 and 50.5 ± 14 years in the ChAdOx1-S vaccine group. Mean body mass index (BMI) value of all enrolled participants was 25.44 kg/m^2^ (± 5.26).

**Prevalence of self-reported adverse events**

We examined the proportion of self-reported local and systemic side effects after each vaccination. A total of 123 (96.85%) participants reported having at least one side effect following vaccination. In general, 112 (88.19%) reported an adverse event of any type at first dose, while 107 (84.25%) and 41 (95.35%) did so at the second and third doses, respectively. The total range of symptoms and frequencies of each symptom in thrice vaccinated participants are presented in Supplementary Table 2. The most commonly reported local side effect was pain at the injection site (63.97%), followed by tenderness (23.23%), and swelling (16.84%). Pain at the injection site was the most common local side effect overall after either the first (65.35%), second (62.99%) or third dose (62.79%) of each vaccine (Supplementary Table 2 and Supplementary Figure 1). The most common systemic side effect was fatigue (47.81%), followed by headache (28.62%), myalgia (25.93%), and fever (22.90%), whereas headache and fever were notably reported after either vaccine dose (Supplementary Table 2 and Supplementary Figure 1). There were also few cases reporting the experience of other adverse events such as chills, bone pain, diarrhea, tachycardia, development of metallic taste in mouth, mild lymphadenopathy, eye rubbing, allergic reaction and emergence of HSV. The overall sum of reported adverse events (N = 811) is higher than the total number of enrolled participants (N = 127) due to the fact that the vast majority of individuals reported more than one reactions simultaneously (total average of adverse reactions = 2.74). The vast majority of symptoms were mild to moderate in terms of severity, in agreement with published reports on vaccine safety.

**Risk factors associated with adverse events of COVID-19 vaccines**

In an exploratory analysis, we assessed the univariate analysis of association between reporting of side effects with participants’ demographic and clinical characteristics. Fisher’s exact test revealed that there were not statistically significant risk factors for adverse reaction onset post vaccination (P values > 0.05; Supplementary Table 3).

Next, we focused on each side effect independently and we assessed age as an associated risk factor for the appearance of post vaccination adverse events. Using the Chi-square test, a statistically significant difference in the prevalence of injection site pain (P < 0.001), fatigue (P = 0.003), fever (P = 0.008), headache (P = 0.001), and nausea (P = 0.026), between the ≤ 45-year-old group and the > 45-year-old group was shown, with younger adults more frequently affected (Supplementary Table 4). Regarding the total number of side effects per participant, it was also significantly higher (P < 0.001) in the ≤ 45-year-old group (4.94 ± 2.44) than in the > 45-year-old group (3.18 ± 2.44) (Supplementary Table 4).

**Blood and serum collection and isolation of peripheral blood mononuclear cells (PBMCs)**

For serology experiments, whole blood was collected in serum separator tubes (SST II Advance Plus Blood Collection Tubes, BD Biosciences). SSTII advance tubes were centrifuged at 1000 × g for 10 min; then, serum was removed from the upper portion of the tube; aliquoted and stored at -80 °C until further use. Where appropriate, serum was heat-inactivated at 56 °C prior use. For IGRA and Milliplex analysis, whole blood was collected in lithium heparin tubes and processed within 30 min from venipuncture.

PBMCs were isolated by density-gradient sedimentation using Lymphosep (Biowest). Isolated PBMCs were cryopreserved in cell recovery media containing 10% dimethyl sulfoxide (DMSO) (AppliChem) and 90% FBS, at a density of 1.0 – 2.0 × 10^7^ cells/mL and stored in liquid nitrogen until use.

**ELISA assays**

All sera were tested for the analysis of vaccine-induced IgG and IgA antibody responses against S1 domain of the SARS-CoV-2 Spike protein, using commercial serological ELISA kits from EuroImmun (EUROIMMUN) according to the manufacturer’s instructions. Anti-NCP IgG responses were also measured via ELISA (EUROIMMUN), in order to detect possible unrecognized SARS-CoV-2-infections, as anti-NCP antibodies are not induced by vaccination. Results were expressed as ratio of the sample’s OD_(450 nm)_ to the calibrator’s OD; and values < 0.8 were considered as negative, > 0.8 to < 1.0 consisted the borderline and values > 1.1 were considered as positive. The presence of neutralizing antibodies was assessed using commercial assays, i.e., cPass SARS-CoV-2 Nabs Detection Kit (Genscript) and SARS-CoV-2-NeutraLISA (EUROIMMUN) according to the manufacturers’ instructions. A value of > 30% inhibition was considered positive for neutralizing activity.

For the detection of vaccine-induced anti-RBD IgG responses, the RBD domain of the SARS-CoV-2 Spike protein (RBD, His & Avi Tag, CHO-expressed, GenScript) was utilized in ELISA development. Briefly, flat bottom high-binding 96-well microtiter plates were coated with 0.1 μg RBD and 0.4 μg BSA per well, diluted in 0.05M carbonate-bicarbonate buffer (pH 9.6). Plates were kept overnight at 4 °C. Then, plates were subsequently washed three times with TBS containing 0.05% Tween20, pH 8 (wash solution). Blocking buffer (1% BSA in TBS) was added at 100 μL volume per well and the plates were incubated for 1 h at room temperature. Samples were prepared at dilution of 1:100 in wash solution containing 1% BSA (sample diluent) and added at 100 µL volume per well. Plates were incubated for 2 h and then washed three times with wash solution. Polyclonal rabbit anti-human IgG (DakoCytomation) antibodies, horseradish peroxidase-labelled, at 1:2000 dilution in sample diluent, was added for 1 h. Then, the plates were subjected to three washes with wash solution. Finally, 100 µL of 3,3′,5,5′-Tetramethylbenzidine (TMB) (Thermo Scientific) were added for 5 min for color development before the addition of 100 µL of stop solution (Thermo Scientific). Using 680 Microplate reader (Bio-Rad), optical density was determined at 450 nm (OD_450_). Sera from 60 healthy controls and 60 patients with autoimmune disorders, collected before the pandemic, were considered as controls. The mean OD_450_ value of controls + 3SD, was set as the cut-off value.

For the quantification of IgG subclasses, samples were diluted in sample diluent buffer (dilution of 1:50) and further incubated for 2 h at room temperature. Subsequently, the plates were washed three times with wash solution and next were incubated with rabbit anti-IgG1, IgG2, IgG3 and IgG4 antibodies (Invitrogen) at a dilution of 1:2000 in sample diluent, for 1 h. Then, plates were washed three times and goat anti-rabbit IgG (DakoCytomation) antibody, horseradish peroxidase-labelled, was added at a dilution of 1:2000 in sample diluent. Plates were incubated for 1 h and then were washed thrice with wash solution and then 100 µL of 3,3′,5,5′-Tetramethylbenzidine (TMB) (Thermo Scientific) were added for 15 min. Then 100 µL of stop solution (Thermo Scientific) were added in each well. The optical density was determined at 450 nm (OD_450_). The mean OD_450_ value of controls + 3SD, was set as the cut-off value for each IgG subclass.

**IGRA assay**

The T-cell response was examined using an Interferon γ release assay (IGRA; EUROIMMUN Medizinische Labordiagnostica AG, Lubeck, Germany), according to the manufacturer’s instructions. Specifically, 500 µL of whole blood, were placed into three individual tubes containing: a) no activating components for the determination of individual background IFNγ levels, b) components of the S1 domain of the Spike protein and c) mitogen, as a non-specific positive control of cell stimulation. After a 24 h stimulation at 37 °C, plasma was harvested from each tube and was evaluated for IFNγ levels on an IFNγ ELISA plate (EUROIMMUN), according to manufacturer’s instructions. Background signals from negative controls were subtracted and final results were calculated in mIU/mL using standard curves. Specifically, values of > 200 mIU/mL were considered as reactive.

**Flow cytometry**

For all flow cytometry assays of stimulated T cells, cryopreserved cells were thawed by warming frozen cryovials in a 37 °C water bath and diluting them in 10 mL pre-warmed complete RPMI-1640 medium containing 10% FBS, 2 mM L-Glutamine, 100 U/mL Penicillin and 100 µg/mL Streptomycin (cRPMI), spun at 350 × g for 7 min. Supernatants were carefully removed, and cells were resuspended in warm cRPMI, counted and apportioned in fresh cRPMI at a density of 5 x 10^6^ cells/mL.

Cells were allowed to rest at 37 °C and 5% CO_2_ for 18 h. Then, cells were plated in 96-well round-bottom plates at a density of 1 x 10^6^ cells/200 µL and were stimulated with co-stimulation (anti-human CD28/CD49d, BD Biosciences) and PepMix covering either Spike N-terminal S1 (aa residues 13-685) or C-terminal S2 (aa residues 686-1273) (JPT Peptide Technologies), for 6 h, at a final concentration of 1 μg/mL/peptide. Matched unstimulated samples from each donor at each time-point were treated with co-stimulation alone in DMSO-containing medium. After stimulation, cells were washed once with DPBS and then stained with Fixable Viability Stain 780 (BD Biosciences) for 15 minutes at room temperature in the dark. Then, cells were pelleted in 96-well V-bottom plates and were stained with an antibody mixture including anti-CD3–BB700 (clone SK7), anti-CD4-PE-Cy7 (clone SK3), anti-CD8-FITC (clone RPA-T8), anti-CD95-APC (clone DX2), anti-CXCR5-BV421 (clone RF8B2), anti-CD45RO-PE (clone UCHL1), anti-CD62L-BV786 (clone SK11), anti-CCR7-BV510 (clone 2-L1-A) in cell staining buffer (DPBS – 1% FBS) for 30 minutes at 4 °C in the dark. At the end of incubation period, cells were washed once and re-suspended in staining buffer prior data acquisition.

For intracellular cytokine staining (ICS), PBMCs were incubated with peptide pools or DMSO-containing medium as described above in the presence of anti-CD107a BV786 (clone H4A3), GolgiStop (BD) and GolgiPLug (BD). T cells exposed to phorbol myristate acetate (PMA; 5 μg/mL, Peprotech) and ionomycin (5 μg/mL, Peprotech) served as positive control. After 6 h of incubation, cells were harvested and stained for viability and surface markers (anti-CD3-BB700, anti-CD4-PE-Cy7, anti-CD8-FITC, all BD Biosciences) in cell staining buffer (DPBS supplemented with 1% FBS) for 30 minutes at 4 °C. Afterwards, samples were fixed and permeabilized using the Cytofix/Cytoperm kit according to manufacturer’s instructions (BD Biosciences). ICS was performed in Perm/Wash buffer using anti-TNF-APC (clone MAb11), anti-IFNγ-PE (clone B27), anti-IL-2-BV510 (clone MQ1-17H12) for 30 minutes at 4 °C. At the end of incubation period, cells were washed with Perm/Wash buffer and re-suspended in staining buffer prior data acquisition.

In all cases, samples were acquired on a FACSMelody cell sorter (BD Biosciences). Compensation was performed using BD CompBeads. Up to 100,000 events were acquired per sample and analyzed with FlowJo software v10.0. S1- and S2-specific immune responses were corrected for background by subtraction of values obtained with DMSO-containing medium. Negative values were set to zero.

**Supplementary References**

1. Menni C, Klaser K, May A, et al. Vaccine side-effects and SARS-CoV-2 infection after vaccination in users of the COVID Symptom Study app in the UK: a prospective observational study. The Lancet Infectious Diseases 2021; 21:939-49.

2. Beatty LA, Peyser DN, Butcher EX, et al. Analysis of COVID-19 Vaccine Type and Adverse Effects Following Vaccination. JAMA Network Open 2021; 4(12):e2140364.

3. Riad A, Pokorná A, Attia S, et al. Prevalence of COVID-19 Vaccine Side Effects among Healthcare Workers in the Czech Republic. Journal of Clinical Medicine 2021; 10, 1428.
