# Supplementary material for: Antibody and T-Cell Subsets Analysis Unveils an Immune Profile Heterogeneity Mediating Long-term Responses in Individuals Vaccinated Against SARS-CoV-2
Source: J Infect Dis. 2022 Oct 19;227(3):353–63. doi: 10.1093/infdis/jiac421 (PMC9620767; doi:10.1093/infdis/jiac421)
Supplement: jiac421_Supplementary_Data [file jiac421_supplementary_data.zip › Agallou_Maria_Supplementary Figure 1.docx]

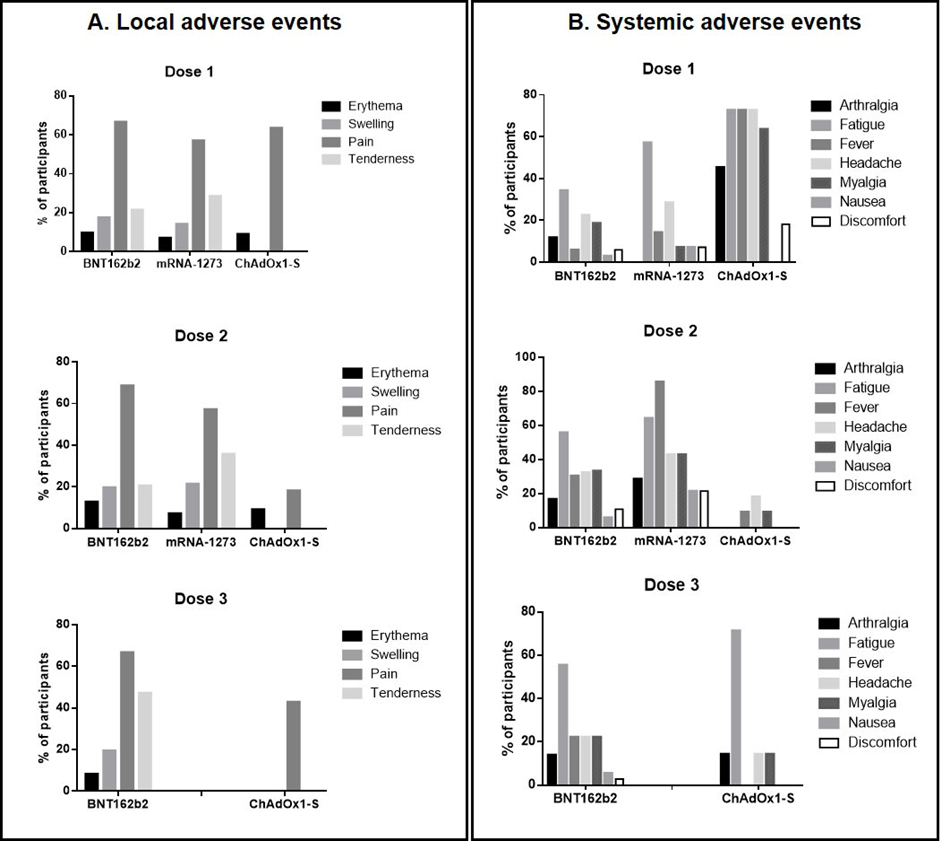


**Supplementary Figure 1.** Prevalence of local (A) and systemic (B) adverse events reported upon receiving the first, second or third dose of different COVID-19 vaccines**.**
