# Supplementary material for: Antibody and T-Cell Subsets Analysis Unveils an Immune Profile Heterogeneity Mediating Long-term Responses in Individuals Vaccinated Against SARS-CoV-2
Source: J Infect Dis. 2022 Oct 19;227(3):353–63. doi: 10.1093/infdis/jiac421 (PMC9620767; doi:10.1093/infdis/jiac421)
Supplement: jiac421_Supplementary_Data [file jiac421_supplementary_data.zip › Agallou_Maria_Supplementary Figure 10_Version_2.docx]

**Supplementary Figure 10.** Analysis of Spike-specific CD4^+^ and CD8^+^ T cell subsets pre-vaccination (Day 0; T_0_) in low (LL) and high (HH) responders. Frequencies of S1 and S2-specifc central memory (CM; CD45RO^+^CD62L^+^CCR7^+^CD95^+^), effector memory (EM; CD45RO^+^CD62L^-^CCR7^-^CD95^+^), stem cell memory (SCM; CD45RO^-^CD62L^+^CCR7^+^CD95^+^) and follicular helper (FH; CD4^+^CXCR5^+^) T cells in CD4^+^ and CD8^+^ T cells are shown. Each dot represents one participant. Horizontal lines indicate mean values. The statistical difference between the two groups is calculated using two-sided Mann-Whitney rank-sum test.
