# Supplementary material for: Antibody and T-Cell Subsets Analysis Unveils an Immune Profile Heterogeneity Mediating Long-term Responses in Individuals Vaccinated Against SARS-CoV-2
Source: J Infect Dis. 2022 Oct 19;227(3):353–63. doi: 10.1093/infdis/jiac421 (PMC9620767; doi:10.1093/infdis/jiac421)
Supplement: jiac421_Supplementary_Data [file jiac421_supplementary_data.zip › Agallou_Maria_Supplementary Figure 11_Version_2.docx]

**Supplementary Figure 11.** Correlation between IFNγ, IL-2, TNFα and IL-13 secretion levels in BNT162b2-vaccinated individuals 7 months post second vaccination. The solid line represents linear regression and error bands represent 95% confidence limits. Spearman’s rank correlation (two-sided) was used to test significance, and p-values and r values (correlation coefficient) are indicated in each panel.
