# Supplementary material for: Antibody and T-Cell Subsets Analysis Unveils an Immune Profile Heterogeneity Mediating Long-term Responses in Individuals Vaccinated Against SARS-CoV-2
Source: J Infect Dis. 2022 Oct 19;227(3):353–63. doi: 10.1093/infdis/jiac421 (PMC9620767; doi:10.1093/infdis/jiac421)
Supplement: jiac421_Supplementary_Data [file jiac421_supplementary_data.zip › Agallou_Maria_Supplementary Figure 2.docx]

**Supplementary Figure 2.** Kinetic analysis of A) anti-S1 IgG antibody responses and B) neutralization activity after ChAdOx1-nCoV-19 vaccination. Serum samples from ChAdOx1-nCoV-19-vaccinated individuals were collected at 1 and 3 months post priming (p.p.) dose and 3 weeks post booster (p.b.) dose. Dashed lines denote the detection cut-off.
