# Supplementary material for: Antibody and T-Cell Subsets Analysis Unveils an Immune Profile Heterogeneity Mediating Long-term Responses in Individuals Vaccinated Against SARS-CoV-2
Source: J Infect Dis. 2022 Oct 19;227(3):353–63. doi: 10.1093/infdis/jiac421 (PMC9620767; doi:10.1093/infdis/jiac421)
Supplement: jiac421_Supplementary_Data [file jiac421_supplementary_data.zip › Agallou_Maria_Supplementary Figure 3_Version_2.docx]

**
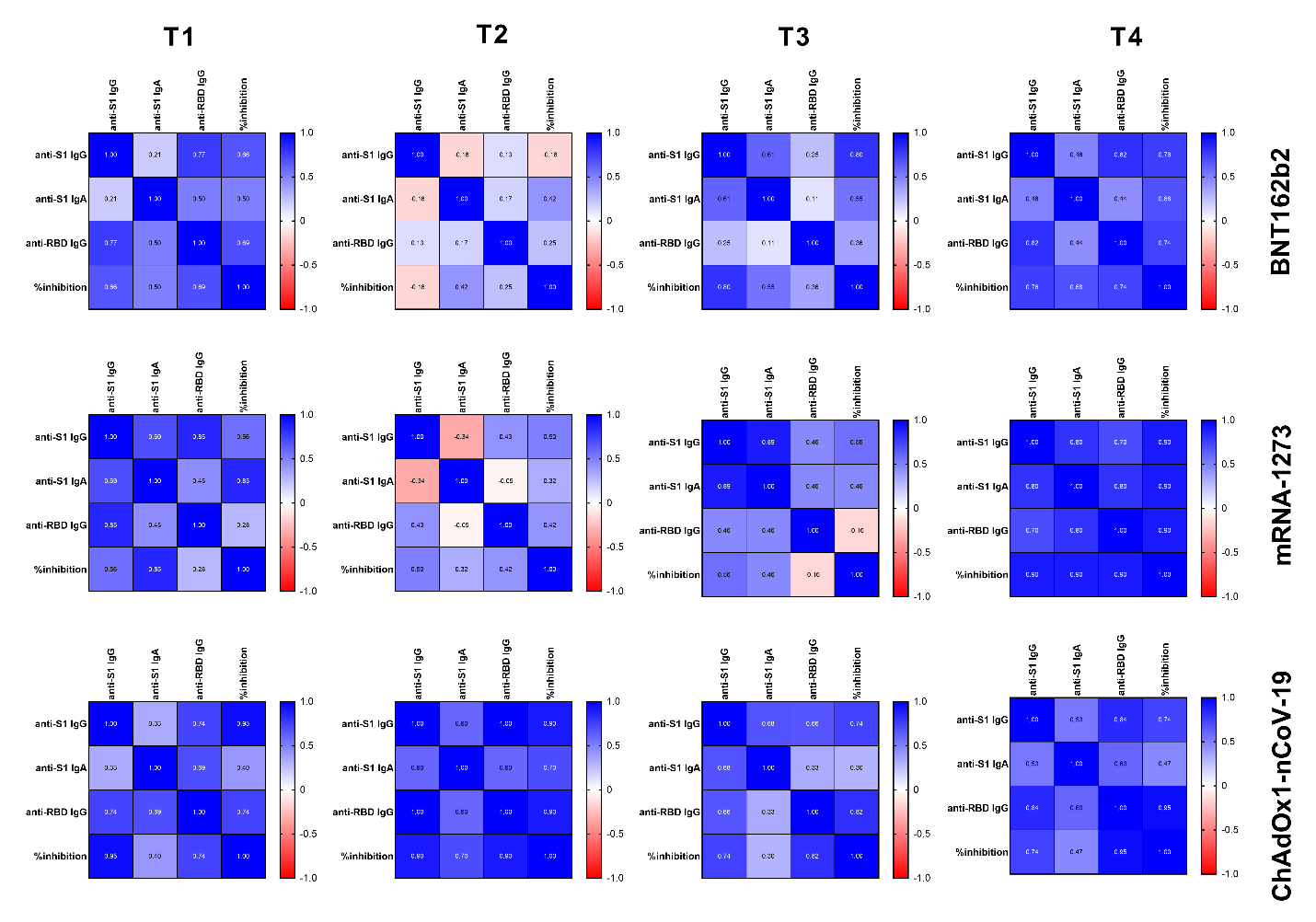
**

**Supplementary Figure 3**. Correlation matrix of anti-S1 IgG titers (Ratio), anti-S1 IgA titers (Ratio), anti-RBD IgG (OD_450_) and neutralization activity (% inhibition) in BNT162b2, mRNA-1273 and ChAdOx1-nCoV-19-vaccinated individuals at T1 (20 days, 4 weeks or 12 weeks, respectively), T2 (20 days), T3 (3 months) and T4 (7 months) post vaccination. Spearman correlation co-efficient is shown.
