# Supplementary material for: Antibody and T-Cell Subsets Analysis Unveils an Immune Profile Heterogeneity Mediating Long-term Responses in Individuals Vaccinated Against SARS-CoV-2
Source: J Infect Dis. 2022 Oct 19;227(3):353–63. doi: 10.1093/infdis/jiac421 (PMC9620767; doi:10.1093/infdis/jiac421)
Supplement: jiac421_Supplementary_Data [file jiac421_supplementary_data.zip › Agallou_Maria_Supplementary Figure 4.docx]

**

Supplementary Figure 4.** Correlation between neutralization activity (% inhibition) and age in BNT162b2, mRNA-1273 and ChAdOx1-nCoV-19-vaccinated individuals at T1 (20 days, 4 weeks or 12 weeks, respectively), T2 (20 days), T3 (3 months) and T4 (7 months). The solid line represents linear regression and the error bands represent 95% confidence limits. Spearman’s rank correlation (two-sided) was used to test significance; p-values and r values (correlation coefficient) are indicated in each panel.
