# Supplementary material for: Antibody and T-Cell Subsets Analysis Unveils an Immune Profile Heterogeneity Mediating Long-term Responses in Individuals Vaccinated Against SARS-CoV-2
Source: J Infect Dis. 2022 Oct 19;227(3):353–63. doi: 10.1093/infdis/jiac421 (PMC9620767; doi:10.1093/infdis/jiac421)
Supplement: jiac421_Supplementary_Data [file jiac421_supplementary_data.zip › Agallou_Maria_Supplementary Figure 6 _Version_2.docx]

**Supplementary Figure 6.** Kinetic analysis of anti-NCP IgG antibody responses in BNT162b2-vaccinated low (LL) and high (HH) responders. Serum samples were collected at 20 days post the priming dose (T1), 20 days (T2), 3 months (T3) and 7 months (T4) after the second dose. Each dot represents one participant. Horizontal lines indicate median values.
