# Supplementary material for: Antibody and T-Cell Subsets Analysis Unveils an Immune Profile Heterogeneity Mediating Long-term Responses in Individuals Vaccinated Against SARS-CoV-2
Source: J Infect Dis. 2022 Oct 19;227(3):353–63. doi: 10.1093/infdis/jiac421 (PMC9620767; doi:10.1093/infdis/jiac421)
Supplement: jiac421_Supplementary_Data [file jiac421_supplementary_data.zip › Agallou_Maria_Supplementary Figure 8_Version_2.docx]

**Supplementary Figure 8.** Analysis of S2-specific CD4^+^ and CD8^+^ T cell subsets in low (LL) and high (HH) reponders at 20 days (T2) and 7 months (T4) post second vaccination. (A) Frequencies of S2-specific IFNγ, IL-2 and TNFα-producing CD4^+^ and CD8^+^ T cells subsets. (B) Polyfunctional analysis and relative distribution of single or multiple cytokine responses in CD4^+^ and CD8^+^ T cells subsets. (C) Frequencies of central memory (CM; CD45RO^+^CD62L^+^CCR7^+^CD95^+^), effector memory (EM; CD45RO^+^CD62L^-^CCR7^-^CD95^+^) and stem cell memory (SCM; CD45RO^-^CD62L^+^CCR7^+^CD95^+^) in CD4^+^ and CD8^+^ T cells. (D) Frequencies of follicular helper (FH; CD4^+^CXCR5^+^) T cells. Each dot represents one participant. Horizontal lines indicate mean values. The statistical difference between the two groups is calculated using two-sided Mann-Whitney rank-sum test.
