# Supplementary material for: Antibody and T-Cell Subsets Analysis Unveils an Immune Profile Heterogeneity Mediating Long-term Responses in Individuals Vaccinated Against SARS-CoV-2
Source: J Infect Dis. 2022 Oct 19;227(3):353–63. doi: 10.1093/infdis/jiac421 (PMC9620767; doi:10.1093/infdis/jiac421)
Supplement: jiac421_Supplementary_Data [file jiac421_supplementary_data.zip › Agallou_Maria_Supplementary Table 1.docx]

**Supplementary Table 1.** Enrolled study participants vaccinated against SARS-CoV-2

| **Reported Vaccine** | **N** | **N1**  **(1 dose reporting)** | **N2**  **(2 doses reporting)** | **N3**  **(3 doses reporting)** |
| --- | --- | --- | --- | --- |
| Pfizer BioNTech (BNT162b2) | 102 | 102 | 102 | 36 |
| Moderna  (mRNA-1273) | 14 | 14 | 14 | 0 |
| Oxford/AstraZeneca (ChAdOx1-S) | 11 | 11 | 11 | 7 |
| **Total** | **127** | **127** | **127** | **43** |
