# Supplementary material for: Antibody and T-Cell Subsets Analysis Unveils an Immune Profile Heterogeneity Mediating Long-term Responses in Individuals Vaccinated Against SARS-CoV-2
Source: J Infect Dis. 2022 Oct 19;227(3):353–63. doi: 10.1093/infdis/jiac421 (PMC9620767; doi:10.1093/infdis/jiac421)
Supplement: jiac421_Supplementary_Data [file jiac421_supplementary_data.zip › Agallou_Maria_Supplementary Table 2.docx]

**Supplementary Table 2.** Prevalence of adverse events to different SARS-CoV-2 vaccines after the first, second or third dose.

| **Variable** | **Side effect** | **BNT162b2** | | | **mRNA-1273** | | **ChAdOx1-S** | | | **TOTAL** |
| --- | --- | --- | --- | --- | --- | --- | --- | --- | --- | --- |
|  |  | **1^st^**  (N = 102) | **2^nd^**  (N = 102) | **3^rd^**  (N = 36) | **1^st^**  (N = 14) | **2^nd^**  (N = 14) | **1^st^**  (N = 11) | **2^nd^**  (N =11) | **3^rd^**  (N = 7) | **N= 297** |
| **Local** | **Erythema** | 10 | 13 | 3 | 1 | 1 | 1 | 1 | 0 | 30 |
|  | **Swelling** | 18 | 20 | 7 | 2 | 3 | 0 | 0 | 0 | 50 |
|  | **Pain** | 68 | 70 | 24 | 8 | 8 | 7 | 2 | 3 | 190 |
|  | **Tenderness** | 22 | 21 | 17 | 4 | 5 | 0 | 0 | 0 | 69 |
| **Systemic** | **Arthralgia** | 12 | 17 | 5 | 0 | 4 | 5 | 0 | 1 | 44 |
|  | **Fatigue** | 35 | 57 | 20 | 8 | 9 | 8 | 0 | 5 | 142 |
|  | **Fever** | 6 | 31 | 8 | 2 | 12 | 8 | 1 | 0 | 68 |
|  | **Headache** | 23 | 33 | 8 | 4 | 6 | 8 | 2 | 1 | 85 |
|  | **Myalgia** | 19 | 34 | 8 | 1 | 6 | 7 | 1 | 1 | 77 |
|  | **Nausea** | 3 | 6 | 2 | 1 | 3 | 0 | 0 | 0 | 15 |
|  | **Discomfort** | 6 | 11 | 1 | 1 | 3 | 2 | 0 | 0 | 17 |
| **Other** | **Other**  (e,g, chills, bone pain, diarrhea, tachycardia) | 4 | 8 | 4 | 1 | 1 | 0 | 0 | 1 | 24 |
| **Mean number per participant** | **(1 – 12)** | 2.2 | 3.1 | 3.0 | 2.4 | 4.4 | 4.2 | 0.6 | 2.0 | 811  2.74 |
