# Supplementary material for: Antibody and T-Cell Subsets Analysis Unveils an Immune Profile Heterogeneity Mediating Long-term Responses in Individuals Vaccinated Against SARS-CoV-2
Source: J Infect Dis. 2022 Oct 19;227(3):353–63. doi: 10.1093/infdis/jiac421 (PMC9620767; doi:10.1093/infdis/jiac421)
Supplement: jiac421_Supplementary_Data [file jiac421_supplementary_data.zip › Agallou_Maria_Supplementary Table 3.docx]

**Supplementary Table 3:** Univariate analysis of association of the reported COVID-19 vaccine adverse events with participants’ demographic and clinical characteristics.

| **Variables** | **Symptomatic** | **Asymptomatic** | **Total** | **Odd Ratio** | **95% CI** | | **p value** |
| --- | --- | --- | --- | --- | --- | --- | --- |
|  | **N = 123** | **N = 4** | **N =127** |  | **Lower bound** | **Upper bound** |  |
| **Sex** | | | | | | | |
| Male | 43 (95.55%) | 2 (4.45%) | 45 (35.43%) | 0.538 | 0.073 | 3.951 | 0.614 |
| Female | 80 (97.56%) | 2 (2.44%) | 82 (64.57%) |  |  |  |  |
| **Age** | | | | | | | |
| Young-middle aged adults  (≤ 45 yrs) | 66 (98.51%) | 1 (1.49%) | 67 (52.76%) | 3.474 | 0.352 | 34.328 | 0.343 |
| Old adults  (> 45 yrs) | 57 (95.00%) | 3 (5.00%) | 60 (47.24%) |  |  |  |  |
| **Blood type** | | | | | | | |
| 0 | 35 (94.59%) | 2 (5.41%) | 37 (29.13%) | 0.398 | 0.054 | 2.935 | 0.579 |
| Other (A, B, AB, unknown) | 88 (97.78%) | 2 (2.22%) | 90 (70.87%) |  |  |  |  |
| **BMI** | | | | | | | |
| Healthy range  (18.5 – 25 kg/ m^2^) | 63 (95.45%) | 3 (4.55%) | 66 (51.97%) | 0.350 | 0.035 | 3.458 | 0.620 |
| Overweight  (> 25 kg/ m^2^) | 60 (98.36%) | 1 (1.64%) | 61 (48.03%) |  |  |  |  |
| **Comorbid condition** | | | | | | | |
| Underlying diseases | 40 (95.24%) | 2 (4.76%) | 42 (33.07%) | 0.482 | 0.065 | 3.547 | 0.599 |
| No diseases | 83 (97.65%) | 2 (2.35%) | 85 (66.93%) |  |  |  |  |
| **Antecedent Covid-19 infection** | | | | | | | |
| Yes | 2 (100.0%) | 0 (0.0%) | 2 (1.57%) | 1.033 | 1.001 | 1.067 | 1.000 |
| No | 121 (96.80%) | 4 (3.20%) | 125 (98.43%) |  |  |  |  |

CI: confidence interval, significant association (unadjusted p < 0.05).
