# Supplementary material for: Antibody and T-Cell Subsets Analysis Unveils an Immune Profile Heterogeneity Mediating Long-term Responses in Individuals Vaccinated Against SARS-CoV-2
Source: J Infect Dis. 2022 Oct 19;227(3):353–63. doi: 10.1093/infdis/jiac421 (PMC9620767; doi:10.1093/infdis/jiac421)
Supplement: jiac421_Supplementary_Data [file jiac421_supplementary_data.zip › Agallou_Maria_Supplementary Table 4.docx]

**Supplementary Table 4:** Prevalence of self-reported local and systemic adverse events in association with median age.

| **TOTAL** | **Variable** | **Outcome** | **≤ 45 Years** | **> 45 Years** | **Total** | **Significance** |
| --- | --- | --- | --- | --- | --- | --- |
|  | **Local side effect** | **Erythema** | 13 (19.40%) | 7 (11.67%) | 20 (15.75%) | 0.232 |
|  |  | **Injection site swelling** | 21 (31.34%) | 11 (18.33%) | 32 (25.20%) | 0.092 |
|  |  | **Injection site pain** | 59 (88.06%) | 36 (60.00%) | 95 (74.80%) | **< 0.0001** |
|  |  | **Tenderness to touch** | 20 (29.85%) | 17 (28.33%) | 37 (29.13%) | 0.851 |
|  | **Systemic side effect** | **Arthralgia** | 19 (28.36%) | 12 (20.00%) | 31 (24.41%) | 0.274 |
|  |  | **Fatigue** | 55 (82.09%) | 35 (58.33%) | 90 (70.87%) | **0.003** |
|  |  | **Fever** | 37 (55.22%) | 19 (31.67%) | 56 (44.09%) | **0.008** |
|  |  | **Headache** | 40 (59.70%) | 18 (30.00%) | 58 (45.67%) | **0.001** |
|  |  | **Myalgia** | 34 (50.75%) | 22 (36.67%) | 56 (44.09%) | 0.111 |
|  |  | **Nausea** | 10 (14.93%) | 2 (3.33%) | 12 (9.45%) | **0.026** |
|  |  | **Discomfort** | 14 (20.90%) | 6 (10.00%) | 20 (15.75%) | 0.092 |
|  | **Other** | **Other**  (e,g, chills, bone pain, diarrhea, tachycardia) | 9 (13.43%) | 6 (10.00%) | 15 (11.81%) | 0.550 |
|  | **Number of side effects** | **(1 – 12)** | 4..94 ± 2.44 | 3.18 ± 2.44 | 4.11 ± 2.59 | **< 0.0001** |
|  | **TOTAL** |  | 67 (52.76%) | 60 (47.24%) | 127 (100%) |  |

Chi-square test and Student’s t-test were used with a significance level of <0.05. Adverse events with statistically significant differences between groups are marked in grey.
